# The interaction between ferroptosis and lipid metabolism in cancer

**DOI:** 10.1038/s41392-020-00216-5

**Published:** 2020-06-30

**Authors:** Dingshan Li, Yongsheng Li

**Affiliations:** 1 Clinical Medicine Research Center, Xinqiao Hospital, Army Medical University, Chongqing, 400037 China; 2 Department of Medical Oncology, Chongqing University Cancer Hospital, Chongqing, 400030 China

**Keywords:** Cancer, Cancer

## Abstract

Ferroptosis is a new form of programmed cell death characterized by the accumulation of iron-dependent lethal lipid peroxides. Recent discoveries have focused on alterations that occur in lipid metabolism during ferroptosis and have provided intriguing insights into the interplay between ferroptosis and lipid metabolism in cancer. Their interaction regulates the initiation, development, metastasis, therapy resistance of cancer, as well as the tumor immunity, which offers several potential strategies for cancer treatment. This review is a brief overview of the features characterizing the interaction between ferroptosis and lipid metabolism, and highlights the significance of this interaction in cancer.

## Introduction

Death is the inevitable fate of life. To date, several types of cell death, including apoptosis, autophagy, necroptosis, pyroptosis, and ferroptosis have been described.^1–4^ Ferroptosis, a unique form of programmed cell death, is characterized by the accumulation of iron-dependent lethal lipid peroxides (LPO).^4^ It is well known that lipid metabolism plays an extremely important role in various tumor properties, including tumorigenesis, invasion, and metastasis.^5^ For example, high saturation levels of membrane lipids protect cancer cells from damage induced by reactive oxygen species (ROS), since saturated membrane lipids are less sensitive to peroxidation.^6,7^ More importantly, ferroptosis is driven by lipid peroxidation,^4^ and LPO are also involved in cell signaling events as intermediates for the synthesis of eicosanoids,^8^ which regulate cell proliferation, survival, invasion, and migration.^9^ In addition, the arachidonoyl (AA) and adrenoyl (AdA) phospholipids can be oxidized to produce LPO by the catalysis of acyl-CoA synthetase long-chain family member 4 (ACSL4) and 15-lipoxygenase (15-LOX/ALOX15).^10,11^ Therefore, the regulation of lipid metabolism and ferroptosis offers several potential strategies for treating cancer. In this review, we will discuss the interaction between ferroptosis and lipid metabolism in cancer.

## Features of ferroptosis

The term ferroptosis was first coined by Dr. Brent R Stockwell in 2012, to describe a unique form of cell death that results from the overwhelming iron-dependent accumulation of lethal amounts of ROS.^4^ Morphologically, the ferroptotic cells exhibit smaller mitochondria, diminished or vanished of mitochondria crista, and condensed mitochondrial membrane densities.^4,10,12^ Compared with camptothecin-induced apoptosis, the nuclear structure of the ferroptotic tumor cells is intact without karyorrhexis and margination of chromatin.^13^ These features are essential for distinguishing ferroptosis from apoptosis, pyroptosis, autophagy, and necrosis.

Intracellular iron and the accumulation of LPO are the fundamentals for ferroptosis. Fe^3+^ is imported by transferrin receptor 1 (TFR1) and deposited in the endosome where Fe^3+^ is converted to Fe^2+^. Subsequently, Fe^2+^ is released from the endosome into a labile iron pool (LIP) in the cytoplasm through divalent metal transporter 1 (DMT1). Excessive iron is stored in ferritin, an iron storage protein complex including ferritin light chain (FTL) and ferritin heavy chain 1 (FTH1).^14–16^ It has been reported that heat shock protein beta-1 (HSPB1) is a negative regulator of ferroptosis as it inhibits the accumulation and absorption of intracellular iron by inhibiting TFR1 expression.^17–20^ Silencing of iron-responsive element binding protein 2 (IREB2), a master transcription factor of iron metabolism, significantly limits erastin-induced ferroptosis.^4^ However, the exact role of iron in ferroptosis remains largely unknown.

**Fig. 1:**
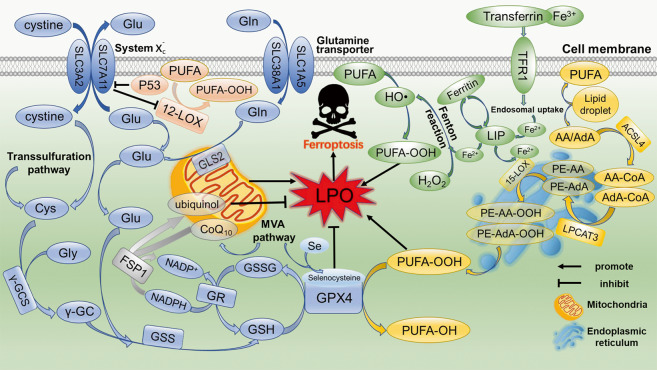
Mechanisms of ferroptosis. The excessive production and failure of elimination of LPO are key causes of ferroptosis. The pathways of eliminating LPO includes system ${\rm{x}}^{\mbox{-}}_{\rm{c}}$/GSH/GPX4 axis and NADPH/FSP1/CoQ_10_ axis. The cystine ingested by system ${\rm{x}}^{\mbox{-}}_{\rm{c}}$ is catalyzed to GSH by γ-GCS and GSS. GPX4 converts GSH to GSSH to reduce LPO and inhibit ferroptosis. FSP1 catalyzes CoQ_10_ to ubiquinol by NADPH, which acts as a lipophilic radical scavenger that reduces LPO. The PUFA-OOH is the main source of LPO. Main peroxidation target PUFAs are AA/AdA mainly present in the endoplasmic reticulum compartment. After catalyzed by ACSL4, LPCAT3, and 15-LOX, AA/AdA is converted to PE-AA-OOH/PE-AdA-OOH to promote ferroptosis. The Fenton reaction mediated by Fe^2+^ produces a large number of HO• to promote the peroxidation of PUFA. P53 transcriptionally inhibits SLC7A11, leading to the production of 12-LOX-mediated PUFU-OOH upon ROS stress. In addition, transsulfuration pathway, MVA pathway and glutaminolysis also participate in the regulation of ferroptosis

Both the impaired elimination and over-production of LPO can cause their accumulation to lethal level during ferroptosis. The cystine availability, glutathione (GSH) biosynthesis, and glutathione peroxidase 4 (GPX4) function are critical for maintaining redox homeostasis and protecting cells from ferroptosis,^4,21,22^ which will be introduced in the following sections in detail (Fig. 1).

## Ferroptosis regulates lipid peroxidation

One of the characteristics of ferroptosis is the accumulation of lethal LPO by lipid peroxidation. ROS are by-products of aerobic metabolism that are continuously produced, transformed, and consumed in all organisms. Superoxide (O_2_^•−^), hydroxyl radical (HO^•^), hydrogen peroxide (H_2_O_2_), and lipid peroxides (ROOH) are common types of ROS in organisms.^23^ ROS possess multiple roles in cancer, such as enhancing cell proliferation and survival, causing DNA damage and genetic instability, cell death and resistance to drugs.^24^ Tumor cells express high levels of antioxidant proteins to reduce ROS levels and maintain redox homeostasis. When redox homeostasis is disrupted, cell death occurs.^4,24^ Many enzymes, including LOXs, NADPH oxidases and cyclooxygenases (COXs) promote endogenous ROS production in cancer.^24^ However, not all ROS induce ferroptosis, rather lipid peroxidation is the primary cause of ferroptosis, which was confirmed by lipophilic antioxidants.^12,25^

During the process of ferroptosis, the production of LPO is complicated. Firstly, the mitochondria in ferroptotic cells are small and mitochondria-targeted antioxidants can inhibit ferroptosis, indicating a correlation of mitochondria with ferroptosis.^4,10,26^ Mitochondria play an important role in cysteine deprivation-induced ferroptosis. Also, the mitochondrial tricarboxylic acid (TCA) cycle and the electron transport chain (ETC) are required for ferroptosis induced by cysteine deprivation. Of interest, mitochondrial function is dispensable for GPX4 inhibition-induced ferroptosis, and GPX4 inactivation may transfer the signal to specific enzymes responsible for producing LPO.^27^ However, mitochondria-independent ferroptosis also exists. Osteosarcoma cells are unable to form ETC-dependent ROS due to the absence of mitochondrial DNA-encoded transcripts, but these cells remain sensitive to erastin as corresponding mtDNA wildtype cells.^4^ Secondly, AA and AdA are oxidized to LPO under the catalysis of ACSL4, lysophosphatidylcholine acyltransferase 3 (LPCAT3) and 15-LOX. Thirdly, iron may contribute to the production of LPO. On the one hand, iron contributes to the production of lethal LPO *via* the Fenton reaction: OH• is generated from H_2_O_2_ by Fe^2+^ catalysis, which can trigger a chain reaction resulting in LPO attacking proximal polyunsaturated fatty acids (PUFAs).^4,28^ On the other hand, iron is an important component for certain enzymes involved in ferroptosis. For instance, Fe^2+^ can serve as a cofactor of lipoxygenases (LOXs) to catalyze PUFA peroxidation.^29^

There are three phases in lipid peroxidation: initiation, propagation, and termination. During initiation, a hydrogen atom from an allylic carbon, particularly from the membrane PUFAs, is extracted by ROS, reactive nitrogen species (RNS), and reactive lipid species (RLS) to form a lipid radical (L•). During the propagation phase, oxygen reacts with L• to form a peroxyl radical (LOO•) that extracts another hydrogen atom from allylic carbon to form a new L• and lipid peroxide LOOH. When two lipid- or peroxyl radicals reach high enough concentrations to interact with each other or endogenous antioxidants (*i.e*. vitamin E or GSH) resulting in donation of a hydrogen atom to form stable non-radical products, the propagation ends and the termination stage is initiated.^28^

In the process of ferroptosis, LPO is converted to an alkoxyl radical (LO•) in the presence of ferrous iron, which subsequently reacts with an adjacent PUFA to initiate another lipid radical chain reaction. During ferroptosis induced by RSL3, GPX4 cannot eliminate the excessive LPO. The propagation stage of lipid peroxidation is then unable to progress to the termination stage. Accumulation of LPO degrades into hydroxy fatty acids or reactive toxic aldehydes, such as malondialdehyde (MDA) and 4-hydroxy-2-nonenal (4-HNE). These RLS can activate lipid peroxidation, dictate cell signaling events by modifying key proteins, or cause toxicity and initiate cell death cascades.^30^ Taken together, ferroptosis produces large amounts of LPO, thus initiating cell death. However, the role of ferroptosis on other aspects of lipid metabolism awaits further investigation.

## Lipid metabolism regulates ferroptosis

Lipid metabolism is closely related to cell sensitivity to ferroptosis *via* several pathways. Recently, a study employing a genome-wide CRISPR-based genetic screening system, and microarray analysis of ferroptosis-resistant cell lines identified that ACSL4 is required for the induction of ferroptosis by accumulating oxidized cellular membrane phospholipids.^10^ With the catalysis of ACSL4, AA, and AdA produce acyl Co-A derivatives. Next, LPCAT3 esterifies these derivatives into phosphatidylethanolamines (AA-PE and AdA-PE) which are primarily present in the endoplasmic reticulum. Subsequently, 15-LOX (ALOX15) directly oxidizes AA-PE and AdA-PE into lipid hydroperoxides, which serve as ferroptotic signals.^10,11^ Furthermore, ACSL4 and GPX4 double-knockout cells can survive without ferroptosis, while supplementation with exogenous AA/AdA and other long PUFAs rescue the sensitivity to ferroptosis in ACSL4 KO cells.^10^ A recent study showed that GPX4 inhibition is unnecessary for ferroptosis mediated by p53 upon ROS stress. Instead, a 12-LOX-mediated, ACSL4-independent ferroptosis pathway plays an essential role in p53-dependent tumor suppression.^31^ In this pathway, p53 indirectly activates the function of 12-LOX through transcriptional inhibition of SLC7A11, leading to 12-LOX-dependent ferroptosis upon ROS stress.^31^ These findings delineate that both ACSL4/LPCAT3/15-LOX and p53/SLC7A11/12-LOX pathways contribute to lethal LPO production during ferroptosis.

System ${\rm{x}}^{\mbox{-}}_{\rm{c}}$, a glutamate-cystine antiporter located in the plasma membrane, imports cystine, which provides cysteine for the biosynthesis of GSH.^4,32^ GSH is a simple tripeptide consisting of glutamate, cysteine, and glycine, with reactive thiol groups on cysteine, which plays a critical role in the reduction of oxidized intracellular components. It has been reported that the transsulfuration pathway is a source of cysteine for GSH.^33^ Furthermore, when the uptake of cystine is reduced, the transsulfuration pathway will be enhanced to facilitate cell survival.^34^ Thus, the transsulfuration pathway can scavenge LPO by providing cysteine for GSH synthesis, thereby inhibiting ferroptosis. Thus, inhibiting cystine uptake in combination with the blockade of the transsulfuration pathway can efficiently enhance ferroptosis.

GPX4, the only enzyme directly reduces lipid hydroperoxides in biomembranes,^35^ can convert GSH to oxidized glutathione disulfide (GSSG) to reduce LPO and maintain cellular redox homeostasis.^21^ Once the system ${\rm{x}}^{\mbox{-}}_{\rm{c}}$/GSH/GPX4 axis is inhibited, a lethal amount of LPO is accumulated, leading to ferroptosis. For example, erastin, a typical inducer of ferroptosis, directly inhibits the system ${\rm{x}}^{\mbox{-}}_{\rm{c}}$ activity, thus disrupting the redox balance and causing the accumulation of LPO, which leads to ferroptosis.^4^ Furthermore, erastin can upregulate the long noncoding RNA *GABPB1-AS1*, which downregulates GABPB1 protein levels by blocking GABPB1 translation, leading to the reduction of peroxiredoxin-5 peroxidase and the eventual suppression of cellular antioxidant capacity.^36^ Cell death induced by RSL3 shares similar features with ferroptosis induced by erastin, *i.e*. a nonapoptotic, MEK-dependent, and iron-dependent oxidative cell death.^37^ It induces ferroptosis by inhibiting GPX4 function.^21^ Selenocysteine is located at the enzyme active site of the selenoprotein GPX4.^29^ Of note, the isopentenylation of selenocysteine tRNA uses isopentenyl pyrophosphate, a product of the mevalonate (MVA) pathway as a donor, which is required for efficient synthesis of selenoproteins.^38^

Recently, ferroptosis suppressor protein 1 (FSP1), formerly designated apoptosis-inducing factor mitochondria associated 2 (AIFM2), was identified as another effective ferroptosis-resistance factor.^39,40^ After myristoylation, FSP1 is recruited to the plasma membrane where it functions as an oxidoreductase that catalyzes coenzyme Q_10_ (CoQ_10_) (also known as ubiquinone-10) to ubiquinol by NADPH, a lipophilic radical scavenger that reduces LPO. Moreover, CoQ_10_ is an important product of the MVA pathway,^38^ indicating that regulating the MVA pathway is a potential strategy to modulate the process of ferroptosis. Hence, the FSP1/CoQ_10_ axis is an independent parallel pathway that functions cooperatively with the system ${\rm{x}}^{\mbox{-}}_{\rm{c}}$/GSH/GPX4 axis to reduce LPO and inhibit ferroptosis.^39,40^

The processes of lipid synthesis, storage and degradation are finely regulated and associated with ferroptosis. Inhibition of steroyl CoA desaturase 1 (SCD1), an enzyme that catalyzes the rate-limiting step in monounsaturated fatty-acid synthesis, can induce both ferroptosis and apoptosis by decreasing CoQ_10_ and unsaturated fatty acyl chains in membrane phospholipids, and increasing long-chain saturated ceramides.^41^ While the inhibition of β-oxidation can restore the sensitivity of tumor cells to ferroptosis.^42^ Moreover, lipophagy, the degradation of intracellular lipid droplets (LDs) *via* autophagy, promotes ferroptosis induced by RSL3 in hepatocytes by decreasing lipid storage, and subsequent lipid peroxidation.^43^

Specific lipids, such as *cis*-unsaturated fatty acids and free fatty acids, can induce, modulate, or suppress both apoptotic and nonapoptotic cell death pathways.^44–46^ It has been demonstrated that oxidized arachidonic and adrenic phosphatidylethanolamine (PEs) navigate cells to ferroptosis.^11^ The exogenous monounsaturated fatty acids (MUFAs), such as exogenous oleic acid (OA) and palmitoleic acid (POA), inhibit induction of ferroptosis by erastin and RSL3.^29,47^ This inhibition requires MUFA activation by ACSL3 and is independent of LDs formation. During ferroptosis, LPO are produced by oxidation of PUFA-containing phospholipids (PUFA-PLs). The extraction of bis-allylic hydrogen atoms from PUFAs induces lipid peroxidation and is necessary for ferroptosis.^29,47^ Therefore, the abundance and location of PUFAs correlate with the degree of intracellular lipid peroxidation, which determines the sensitivity of cells to ferroptosis. Once exogenous MUFAs are activated by ACSL3, they can displace PUFAs from PLs located at the plasma membrane and reduce the sensitivity of plasma membrane lipids to oxidation.^47^ Furthermore, a series of enzymes mentioned above, such as ACSL4, LPCAT3 and 15-LOX, involved in the regulation of PUFA synthesis and decomposition can regulate the sensitivity of cells to ferroptosis.^10,11^ Therefore, lipid metabolism can regulate ferroptosis and determine the sensitivity of cells to ferroptosis.

## The interaction of ferroptosis and lipid metabolism in cancer biology

**Fig. 2:**
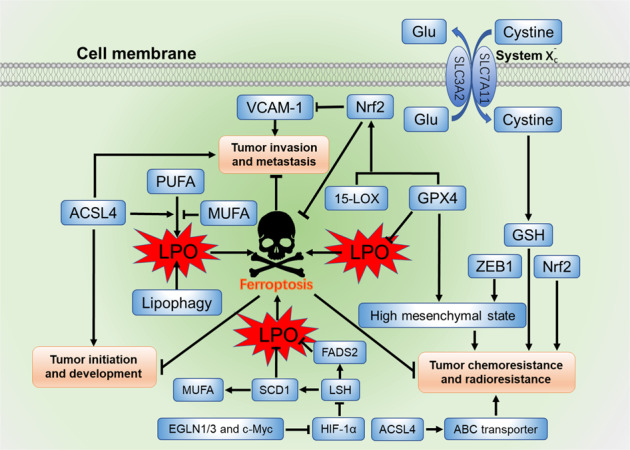
The interaction of ferroptosis and lipid metabolism in tumor biology. ACSL4, 15-LOX, and GPX4 are key factors involved in ferroptosis and lipid metabolism that regulate tumor initiation, development, invasion, metastasis, chemoresistance, and radioresistance. GPX4 and ZEB1 are associated with high mesenchymal state to contribute to tumor chemoresistance. Both GPX4 and 15-LOX can activate Nrf2 to inhibit the expression of VCAM-1 that contribute to tumor metastasis and angiogenesis. The transcription factor Nrf2 is a key regulator of antioxidant response that suppress ferroptosis to enhance tumor chemoresistance and radioresistance. EGLN1/3 and c-Myc can directly activate the expression of LSH by suppressing HIF-1α, and the elevated LSH upregulates genes involved in lipid metabolism, such as SCD1 and FADS2 to suppress ferroptosis and promote tumor initiation and development

Cancer cells often have defects in executing cell death. To facilitate growth, cancer cells require higher levels of iron and lipid metabolism than normal cells, which also makes cancer cells more susceptible to ferroptosis. In recent years, accumulating evidence has elucidated the interaction of ferroptosis and lipid metabolism in the initiation, development, invasion, metastasis, and therapy resistance of cancer (Fig. 2).

### The interaction modulates the initiation and development of cancer

Lipid metabolism regulates ferroptosis during the initiation and development of cancer. For example, lipophagy promotes ferroptosis induced by RSL3 in HepG2 cells by decreasing lipid storage and subsequent lipid peroxidation.^43^ The exogenous lipids can also regulate ferroptosis in tumors. Compared to ω-3 fatty acids, the ω-6 fatty acids are more efficient in inducing ferroptosis in ACSL4-KO Pfa1 cells.^10^ As mentioned above, MUFAs and PUFAs determine the degree of intracellular lipid peroxidation which promotes the sensitivity of cells to ferroptosis.^29,47^ In addition, a recent study showed that SCD1, a crucial enzyme in lipid metabolism that catalyzes the rate-limiting step in MUFAs synthesis, protects ovarian cancer cells from ferroptotic cell death, indicating that lipid metabolism is involved in the activation of ferroptosis.^41^

ACSL4 has been shown to be overexpressed in multiple cancer types.^48–54^ It enriches cellular membranes with long polyunsaturated ω-6 fatty acids and contributes to the production of LPO.^10,55^ Knockdown of ACSL4 decreases the 17β-estradiol-induced cancer cell migration, proliferation, and invasion properties,^56^ while overexpression of ACSL4 enhances tumor growth and proliferation.^48,49,56^ Furthermore, compared with ferroptosis-sensitive cancer cells, the expression of ACSL4 was notably downregulated in ferroptosis-resistant cancer cells.^55^ Hence, upregulation of ACSL4 enhances tumor growth and proliferation but also promotes the sensitivity to ferroptosis.

Hypoxia-inducible factor-1 (HIF-1) is a crucial factor which plays an important role in the development of cancer, and it is well known to regulate glycolysis, lipid metabolism and glutaminolysis.^57,58^ The iron-dependent enzymes Egl-9 family hypoxia-inducible factor 1/3 (EGLN1/3) and c-Myc directly activate the expression of chromatin remodeling factor lymphoid-specific helicase (LSH) by suppressing HIF-1α in lung cancer progression. The elevated LSH upregulates genes involved in lipid metabolism, such as SCD1 and fatty-acid desaturase 2 (FADS2) to suppress ferroptosis by inhibiting the accumulation of LPO and intracellular iron.^59,60^ In addition, HIF-2 selectively enriches lipids that contain polyunsaturated fatty acyl side chains and induces a ferroptosis-susceptible cell state, by activating the expression of hypoxia-inducible, lipid droplet-associated protein (HILPDA).^61^ Hence, upregulating SCD1 and FADS2, the genes involved in lipid metabolism, or downregulating HIF-1α/2α may enable tumor cells to become more resistant to ferroptosis, thus promoting tumorigenesis.

The P53 tumor suppressor is a critical inhibitor in the development of cancer, and is also closely related to ferroptosis. On the one hand, P53 can promote the accumulation of lipid hydroperoxides to enhance ferroptosis by increasing the expression levels of 15-LOX by promoting the expression of spermidine/spermine N1-acetyltransferase1 (STA1), or increasing the expression of GLS2, and activating the function of 12-LOX through transcriptional inhibition of SLC7A11.^31,62,63^ On the other hand, P53 can suppress ferroptosis by decreasing the accumulation of LPO. For instance, P53 directly suppresses the activity of dipeptidyl peptidase 4 (DPP4) and increases the expression of cyclin dependent kinase inhibitor 1A, also known as p21^WAF1/Cip1^ (CDKN1A/p21).^64,65^ These studies indicate that the interaction between ferroptosis and lipid metabolism contributes to P53-mediated tumor suppression.

### The interaction modulates tumor invasion and metastasis

The interaction between ferroptosis and lipid metabolism are also related to tumor invasion and metastasis. First, GPX4, a key regulator of ferroptosis, inhibits the activities of COXs and LOXs by reducing lipid peroxidation level in cells. Overexpression of GPX4 decreases the lung metastatic colonizing capacity of B16BL6 cells. Meanwhile, invading cancer cells are the source of metastasis-promoting eicosanoids. The metastatic ability of GPX4-overexpressing L929 cells was suppressed by reducing eicosanoid production.^66^ Furthermore, both GPX4 and 15-LOX, regulators of ferroptosis, can upregulate endogenous heme oxygenase-1, likely *via* activation of nuclear factor erythroid 2-related factor 2 (Nrf2) to inhibit the expression of vascular cell adhesion molecule-1 (VCAM-1) that contribute to tumor metastasis and angiogenesis.^67^ Overexpression of GPX4 with a subsequent decrease in intracellular phospholipid hydroperoxides causes the down-regulation of irradiation-induced MMP-1.^68^ These studies demonstrate that GPX4 inhibits the invasion and metastasis of tumors. However, when GPX4 is inhibited, the conversion of GSH to GSSH will be blunted and LPO will accumulate to cause ferroptosis in tumor cells.^21^ Secondly, iron is essential for LPO production and ferroptosis. Iron is an important component involved in ferroptosis that facilitate the LOXs catalysis of PUFA peroxidation. The iron chelator Dp44mT and overexpression of ferroportin can inhibit tumor metastasis.^69,70^ Thirdly, ACSL4 and 15-LOX are key regulators in lipid metabolism and producing LPO during ferroptosis, both of which are implicated in the metastasis and angiogenesis of tumors.^71^ For example, 15-LOX suppresses the growth and metastasis of mammary gland and Lewis lung carcinoma in mice.^72^ Moreover, miRNA-17–92 is an oncogenic miRNA cluster that plays an important role in the angiogenesis of tumor^73^ by protecting endothelial cells from erastin-induced ferroptosis *via* targeting ACSL4.^74^ ASCL4-mediated lipid metabolism promotes the invasion and migration in breast and prostate cancer cells,^48,49^ however, LPO produced by ACSL4-mediated lipid metabolism promotes ferroptosis. These findings indicate that ferroptosis is an effective treatment regimen for prohibiting cancer invasion and metastasis.

### The interaction regulates chemoresistance and radioresistance

Chemoresistance and radioresistance are the primary causes of traditional cancer treatment failure. As a new death mode characterized by LPO accumulation, ferroptosis plays an important role in regulating the chemoresistance and radioresistance of tumor.

It has been reported that LPO modulators alone can effectively treat multidrug-resistant cancer.^75^ The system ${\rm{x}}^{\mbox{-}}_{\rm{c}}$/GSH/GPX4 axis also contributes to regulation of tumor chemoresistance. For instance, cells with increased GSH are more resistant to chemical drugs, such as doxorubicin,^76^ cisplatin,^77^ and 5-fluorouracil.^78^ The destruction of the ATP-binding cassette (ABC)-family transporter multidrug resistance protein 1 (MRP1) can inhibit the outflow of GSH and suppress ferroptosis.^79^ Moreover, SLC7A11, the components of system ${\rm{x}}^{\mbox{-}}_{\rm{c}}$, mediates cystine uptake in tumor cells, however, is resistant to geldanamycin by providing cystine for GSH maintenance.^80^ During ferroptosis induced by system ${\rm{x}}^{\mbox{-}}_{\rm{c}}$ inhibition, GSH is decreased. This indicates that enhancing ferroptosis may be an effective intervention strategy for multidrug-resistant tumor cells. The pharmacological and genetic inhibition of system ${\rm{x}}^{\mbox{-}}_{\rm{c}}$ can induce ferroptosis in the cisplatin-resistant head and neck cancer (HNC)^81^ and pancreatic ductal adenocarcinoma (PDAC).^82^ Some chemotherapies are sensitive to ferroptosis induced by GPX4 inhibition.^83,84^ Moreover, as a key regulator of lipid metabolism, ACSL4 also produces LPO during the process of ferroptosis. ACSL4 has been shown to participate in the chemoresistance of breast cancer cells by regulating ABC transporter expression.^85^ Pancreatic cancer is one of the most aggressive gastrointestinal malignancies. Due to its potent chemotherapy resistance and poor prognosis, current chemotherapy drugs showed low therapeutic efficacy. The plant growth regulator cotylenin A (a inducer of ROS) and phenethyl isothiocyanate (a dietary anticarcinogenic compound) have been shown to synergistically inhibit the proliferation of human pancreatic cancer cells, such as MIAPaCa-2 and gemcitabine-resistant PANC-1 cells, by inducing ferroptosis.^86^

During chemotherapy, tumor cells evolve to produce multidrug-resistant tumor cells originated from persister cell pool. Therefore, targeting persister cells can prevent tumor relapse. Persisiter cells are associated with mesenchymal state. It has been found that a high mesenchymal therapy-resistant cell state is dependent on the GPX4 that protects against ferroptosis.^84^ Moreover, vemurafenib, a small molecule targeting mutant BRAF, can induce melanomas dedifferentiation that increases sensitivity to ferroptosis with marked lipidomic changes, including the accumulation of PUFAs.^87^ In addition, a systematic study of cellular responses to ferroptosis inducer revealed that high mesenchymal state is associated with zinc-finger E-box binding homeobox 1 (ZEB1), a transcription factor reported to be a central regulator of lipid metabolism and adipogenic fate and can enhance stemness, colonization capacity, and metabolic plasticity of tumor cells.^83,88,89^ This indicates that the interaction of ferroptosis and lipid metabolism can regulate mesenchymal-like phenotype associated with drug-resistance.

In addition, ferroptosis also affects cancer sensitivity to radiotherapy. Ionizing radiation (IR) induces not only the expression of ACSL4, a lipid metabolism enzyme required for ferroptosis, but also the accumulation of LPO, resulting in ferroptosis. ACSL4 ablation significantly abolishes IR-induced ferroptosis and promotes radioresistance.^90^ In addition, inhibiting 12-LOX can modulate the radiosensitivity of human prostate cancer cells,^91^ suggesting that the P53/12-LOX-mediated, ACSL4-independent ferroptosis pathway may be involved in the regulation of radioresistance. These interaction of ferroptosis and lipid metabolism highlights the potential strategies for sensitizing cancer radiotherapy and chemotherapy.

### Ferroptosis resistance in cancer cells

Cancer cells acquire ferroptosis resistance *via* multiple pathways. For example, ACSL3 and SCD1, the key regulators of lipid metabolism, are required for inducing the ferroptosis-resistant state.^41,47^ But the detailed underlying mechanisms by which lipid metabolism drives ferroptosis-resistance in cancer cells awaits further exploration. The transcription factor Nrf2 is a major regulator of antioxidant response because many of its downstream target genes are involved in maintaining redox homeostasis. Notably, two inducers of ferroptosis, RSL3 and erastin, respectively initiate the ferroptotic cascade through inhibiting glutathione GPX4 and system ${\rm{x}}^{\mbox{-}}_{\rm{c}}$, both of which are the downstream targets of Nrf2. Furthermore, many other proteins and enzymes, such as GPX4, NADPH, FTH1, FTL, and ferroportin, which are responsible for preventing lipid peroxidation and thus cause ferroptosis, are the target genes of Nrf2.^92^ For instance, artesunate has been identified as a specific activator of ferroptosis.^93^ The activation of Nrf2-antioxidant response element (ARE) pathway can decrease artesunate sensitivity and increase the ferroptosis resistance in cisplatin-resistant HNC cells.^94^ The Nrf2-ARE pathway also contributes to the resistance of HNC cells by inhibiting GPX4. Overexpression of Nrf2 enhances HN3 cells resistance to GPX4 inhibitor RSL3 by inhibiting Keap1 or Nrf2 gene transfection. Moreover, Nrf2 inhibition can sensitize HN3R cells to RSL3.^95,96^ Thus, inhibiting Nrf2 pathway can abolish the resistance to ferroptosis in cancer cells. In addition, chemotherapy promotes exosome secretion from stromal cells in the tumor microenvironment, such as cancer-associated fibroblasts, leading to decreased LPO accumulation and ferroptosis in cancer cells.^97^ Thus, reversing the ferroptosis resistance is a potential strategy to improve the efficacy of anti-cancer therapy.

## The interaction of ferroptosis and lipid metabolism modulates tumor immunity

**Fig. 3:**
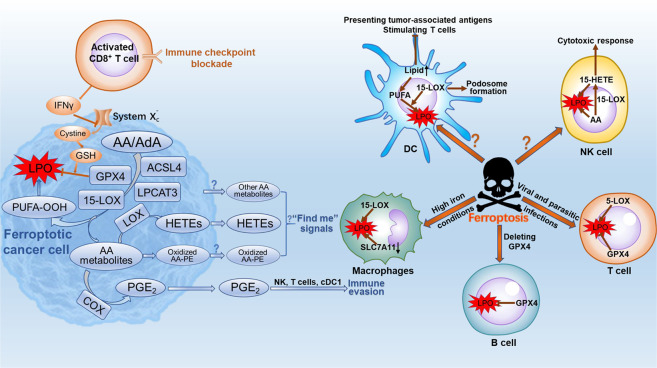
The interaction of ferroptosis and lipid metabolism in modulating tumor immunity. With the catalysis of ACSL4, LPCAT3, and 15-LOX, AA/AdA is oxidized to LPO that initiate ferroptosis. Some AA/AdA metabolites *e.g*., HETEs released from ferroptotic cancer cells activate antitumor immunity, while other lipids *e.g*., PGE_2_ suppress immunity to promote tumor cell evasion. Immune cells also regulate the ferroptosis of cancer cells. Immunotherapy-activated CD8^+^ T cells induce the ferroptosis of cancer cells by releasing IFNγ to downregulate the system ${\rm{x}}^{\mbox{-}}_{\rm{c}}$. Thus, GSH level in tumor cells is not enough to eliminate LPO by GPX4, which leads to ferroptosis. Under certain conditions, immune cells including T cells, B cells, macrophages also undergo ferroptosis, which will modulate the tumor immunity

Dysregulated immunity is a hallmark of cancer. The interaction of ferroptosis and lipid metabolism plays a critical role in modulating tumor immunity (Fig. 3). Firstly, the ferroptotic cells influence immune cells by releasing lipid metabolites. The mobilization of immune cells to, and within, tumors is controlled by various chemokines, cytokines, and soluble metabolites (e.g., lipids and nucleic acids).^98^ However, it is unknown whether ferroptotic cancer cells can produce such signals. Evidence has shown that macrophages clear cells undergoing ferroptosis, support the existence of such signals.^99^ Moreover, the accumulation of lipid droplets has been recognized as a reservoir for AA or other PUFAs that are mobilized for the formation of lipid mediators signals, such as eicosanoids.^100^ Enzyme oxidized phospholipids (eoxPLs) are formed by binding of eicosanoid acids to phospholipid (PLs) in immune cells, and they play an important role in immune regulation in health and diseases. The eoxPLs, such as 15-hydroxyeicosatetraenoic acid (15-HETE), oxidized AA-PE and AdA-PE, which are produced by the ACSL4/LPCAT3/15-LOX axis, promote ferroptosis.^55,101,102^ In addition, the cells release oxidized lipid mediators such as 5-HETE, 11-HETE and 15-HETE during GPX4 deletion-induced ferroptosis.^102^ Hence, AA metabolites may be the potential “find me” signals to recruit immune cells to find ferroptotic cancer cells. The ACSL4/LPCAT3/15-LOX axis not only oxidize AA or AdA to LPO to elicit ferroptosis but also may produce “find me” signals from ferroptotic tumor cells, mediating antitumor immunity. In addition, ferroptotic tumor cells express higher *PTGS2*, a gene encoding COX-2, to produce PGE_2_,^21^ which is a major immunosuppressive factor that dampen the antitumor immunity of conventional type 1 dendritic cells (cDC1), natural killer (NK) cells and cytotoxic T cells.^103–106^

The high mobility group box 1 (HMGB1) is a biomarker of ferroptosis and is also an important factor for cancer immunity.^107,108^ Knockdown of HMGB1 decreases erastin-induced ferroptosis *via* the RAS-JNK/p38 pathway.^109^ HMGB1 released from ferroptotic cancer cells promotes the M1 polarization by the HMGB1-AGER signaling pathway.^108,110^ Moreover, oxidized phospholipids generated by 12/15-LOX are substrates for key proteins required for effective autophagy,^111^ while HMGB1 can be released in an autophagy-dependent manner.^108^ Hence, the interaction of lipid metabolism and ferroptosis may influence the release of HMGB1 thereby modulating tumor immunity. However, the underlying mechanisms remain elusive.

Secondly, the phenotypes and functions of immune cells can be directly influenced by ferroptosis. For example, ferroptotic cancer cells release oncogenic KRAS protein which can be engulfed and drive tumor-associated macrophage polarization.^112^ Ferroptosis also occurs in macrophages.^113–115^ However, compared with cancer cells, macrophages exert higher resistance to ferroptosis. Erastin and deleting SLC7A11 expression were not sufficient to induce macrophage ferroptosis unless under high iron conditions.^114,116^ In addition, compared with M2 macrophages, M1 macrophages exert higher resistance to ferroptosis.^115^ 15-LOX modulates the ferroptotic endurance in macrophages, similar to that in tumor cells,^115,117^ while solely knocking out 12/15-LOX is insufficient to prevent lipid peroxidation in T cells.^11,118^ Instead, 5-LOX may be responsible for toxic lipid peroxidation in T cells lacking GPX4.^118–120^ Hence, during ferroptosis, the change of lipid metabolism in immune cells are different from cancer cells. However, the mechanism by which ferroptosis occurs in macrophages and T cells remain unclear. In addition, CD8^+^ T cells from T cell-specific GPX4-deficient mice are unable to maintain homeostasis in the periphery, and both antigen-specific CD4^+^ and CD8^+^ T cells lacking GPX4 fail to expand. *Ex vivo*, GPX4-deficient T cells rapidly accumulate membrane LPO and undergo ferroptosis.^118^ Moreover, GPX4 is required to prevent lipid peroxidation and ferroptosis in B cells and in diffuse large B-cell lymphoma.^121,122^

DCs in tumor-bearing mice and cancer patients show increased lipid levels, which suppress the presentation of tumor-associated antigens and the function of stimulating T cells.^123^ As reported earlier, the levels of PUFAs determine the degree of lipid peroxidation, and the accumulated lipid droplets serve as a reservoir for PUFAs.^29,47^ Moreover, oxidized lipids and lipid bodies containing oxidative truncated lipids in DCs can inhibit cross-presentation in cancer.^124,125^ The 12/15-LOX–derived lipid mediators limit the maturation process of DCs and dampen the differentiation of Th17 cells.^126^ Inhibition of 15-LOX in DCs was reported to decrease podosome formation, thus suppressing antigen uptake and migration capacity.^127^ In addition, AA and its 15-LOX products, are required for the cytotoxic response of NK cells to tumor target cells.^128^ Hence, understanding the relationship between ferroptosis and immune cells in the tumor microenvironment is important to develop efficient treatment strategies.

Thirdly, immune cells also regulate the ferroptosis of cancer cells. During tumor invasion, the naive CD8^+^ T cells differentiate into effector CD8^+^ T cells, and further activate into cytotoxic CD8^+^ T cells and memory CD8^+^ T cells for their targeted role in the tumor site.^129^ Cancer immunotherapy can reinforce the effect of CD8^+^ T cells in the tumor microenvironment.^130–132^ The cytokines including interferon γ (IFNγ) released by immunotherapy-activated CD8^+^ T cells downregulate the expression of SLC3A2 and SLC7A11, the components of system ${\rm{x}}_{\rm{c}}^{\mbox{-}}$, and reduce cystine uptake by tumor cells.^130^ Thus, GSH in tumor cells is not enough to eliminate LPO by GPX4. The propagation stage of lipid peroxidation cannot be switched into termination stage, the excessive accumulation of LPO leads to ferroptosis. Furthermore, cysteine deprivation combined with checkpoint blockade synergistically improves the efficacy of antitumor immunity mediated by T cells in mice.^130^ Indeed, for the patients with nivolumab therapy, the clinical benefits are positively associated with reduced expression of SLC3A2 and increased IFNγ.^130^ However, despite IFNγ is a well-known antitumor cytokine, it also contributes to tumor escape under certain circumstances.^133^ For example, IFNγ secreted by activated CD8^+^ T cells upregulates PD-L1 on cancer cells and promotes tumor growth.^134^ Thus, combining checkpoint blockade with ferroptosis induced by T cells is a potential therapeutic approach for cancer.

Taken together, ferroptosis is a double-edge sword in modulating tumor immunity. On the one hand, ferroptosis inducers and IFNγ secreted by activated CD8^+^ T cells promote ferroptosis of cancer cells to play an antitumor role. The ferroptotic cancer cells also release specific signals, such as AA metabolites and HMGB1, to mediate antitumor immunity. On the other hand, in order to help adjacent cancer cells to survive from or escape immunity, the ferroptotic cancer cells and tumor-infiltrating immune cells produce immunosuppressive mediators including PGE_2_, or upregulate immune checkpoints to dampen the antitumor immunity and promote tumor growth.

## Clinical trials and preclinical drugs targeting ferroptosis for antitumor treatment

Numerous preclinical studies and clinical trials focusing on ferroptosis have been, and are being, performed (Table 1). For example, sorafenib, a system ${\rm{x}}_{\rm{c}}^{\mbox{-}}$ inhibitor, is used in the treatment of carcinomas from renal, thyroid, and liver.^135–137^ Sulfasalazine induces ferroptosis by inhibiting system ${\rm{x}}_{\rm{c}}^{\mbox{-}}$ and is used in clinical trials for glioma treatment.^135,138^ Altretamine has been applied in ovarian cancer and inhibits GPX4 to induce ferroptosis of ovarian cancer cells.^139,140^ Moreover, neratinib, a tyrosine kinase inhibitor, potently promotes ferroptosis and inhibits the growth and brain metastasis of breast cancer.^141^ Also, ACSL4 is a predictive biomarkers of neratinib response and facilitates the patient selection.^141^ In addition, lapatinib can induce ferroptosis in breast cancer cells by altering iron transport system.^142^ BAY-87–2243 triggers accumulation of LPO and ferroptosis by inhibiting mitochondrial complex I and HIF-1. This drug has reached phase 1 clinical trials for treatment of advanced malignancies.^143^

**Table 1:** Clinical trials and preclinical drugs targeting ferroptosis and lipid metabolism for antitumor treatment

Nos.	Drugs	Target	Clinical use/clinical trials	Refs.
1	Sorafenib	System ${\mathrm{x}}_{\mathrm{c}}^ -$ inhibitor	Renal cell, thyroid, and hepatocellular carcinoma treatment	^135–137^
2	Altretamine	GPX4 inhibitor	Ovarian cancer treatment	^139,140^
3	Buthionine sulfoximine	γ-GCS inhibitor	Clinical trials for neuroblastoma treatment	^21,42,96^
4	Statins	Block biosynthesis of CoQ10	Cholesterol reducing agents	^83^
5	Sulfasalazine	System ${\mathrm{x}}_{\mathrm{c}}^ -$ inhibitor	Rheumatoid arthritis and inflammatory bowel diseases treatment/clinical trials for glioma treatment	^135,138^
6	Lapatinib and neratinib	Kinase inhibitor and alters iron transport system	Clinical trials for solid and metastatic breast cancer treatment	^141,142^
7	BAY-87-2243	Mitochondrial complex I inhibitor/HIF-1 inhibitor	Phase I trial for treatment of advanced malignancies	^143^
8	Salinomycin	Antioxidant properties inhibitor and alters iron transport system	Preclinical trials for breast cancer treatment	^144–146^
9	MF-438	SCD1 inhibitor	Preclinical trials for melanoma, breast and lung cancer treatment	^41,148–150^
10	CAY10566	SCD1 inhibitor	Preclinical trials for breast, prostate and liver cancer treatment	^41,151–153^
11	Aminooxyacetic acid	Mitochondrial fatty-acid synthesis	Preclinical trials for colon cancer treatment	^4,155,156^
12	Triacsin C	ACSL1,3,4 inhibitor	Preclinical trials for cancer treatment	^47,157^
13	thiazolidinediones	ACSL4 inhibitor	Diabetes/clinical trials for cancer treatment	^10,157^
14	Zileuton	5-LOX inhibitor	Clinical trials for lung, head and neck cancer and chronic myelogenous leukemia treatment	^158–161^
15	Baicalein	12/15-LOX inhibitor	Clinical trials for acute lymphocytic leukemia treatment	^29,162^

Cancer stem cells (CSCs) are a subset of tumor cells with the characteristics of self-renewal, differentiation, and tumorigenicity. CSCs are intrinsically resistant to conventional cancer treatment, which is related to relapse and metastasis. Salinomycin is a selective drug that targets CSCs.^144^ Ironomycin (AM5), a synthetic derivative of salinomycin, shows a more potent selective activity against breast CSCs through accumulating and sequestrating of iron in lysosomes, resulting in the accumulation of LPO.^145^ In another study, salinomycin conjugated with biocompatible gold nanoparticles (AuNPs) coated with poly (ethylene glycol) can promote the accumulation of LPO in breast CSCs to cause ferroptosis.^146^ In addition, p53 regulates oxidative stress and the metal-organic network (MON) which contributes to Fenton reaction. A p53 plasmid encapsulated MON (MON-p53) can kill cancer cells through ferroptosis and apoptosis, thereby reducing metastasis.^147^

Since the interaction of ferroptosis and lipid metabolism plays an important role in cancer biology, several small molecules targeting this interaction have been applied in antitumor treatments. For example, MF-438 and CAY10566 promote ferroptosis by inhibiting SCD1.^41^ MF-438 is used in preclinical trials for the treatment of melanoma, breast and lung cancer.^148–150^ CAY10566 is used in preclinical trials for the treatment of breast, prostate and liver cancer.^151–153^ Piperazinylpyridines and nicotinamide or pyridazine derivatives are used in the treatment of cancer by targeting SCD1.^154^ The transaminase inhibitor aminooxyacetic acid can suppress ferroptosis by targeting mitochondrial fatty-acid synthesis and is employed in preclinical trials for colon cancer treatment.^4,155,156^ As mentioned above, ACSL3 is required for inducing the ferroptosis-resistance, while ACSL4 is essential for inducing ferroptosis.^10,47^ Hence, ferroptosis inducer combined with ACSL3 inhibitor may lead to a better antitumor efficacy. Because of the conserved catalytic domain that endows ACSL isoenzymes with redundant catalytic functions, there is no selective ACSL3 inhibitor yet.^157^ But some molecules, such as Triacsin C and thiazolidinedione, showed a potent inhibitory effect on ACSLs and promoted ferroptosiss.^10,47^ Zileuton, a 5-LOX inhibitor, can inhibit ferroptosis and is used in clinical trials for the treatment of head and neck cancer, lung cancer and chronic myelogenous leukemia.^158–161^ Baicalein and PD-146176 rescues cancer cells from erastin-induced ferroptosis by inhibiting 12/15-LOX.^29^ Baicalein is also used in clinical trials for acute lymphocytic leukemia treatment.^162^ Therefore, combined treatment with small molecules targeting the interaction between ferroptosis and lipid metabolism shed new light on the treatment of various cancers.

## Conclusion

Distinct from other forms of cell death, ferroptosis is characterized by the LPO accumulation and iron dependency. Recent studies have provided new insights into the molecular mechanism of ferroptosis, particularly its relationship with lipid metabolism in cancer cells. The interaction between ferroptosis and lipid metabolism plays an important role in tumorigenesis, tumor development, invasion, metastasis, therapy resistance, and tumor immunity. The detailed underlying mechanisms driving ferroptosis awaits further exploration. We should address the specific markers for ferroptosis, the specific source and role of LPO in ferroptosis, as well as the exact interaction between ferroptosis and lipid metabolism in cancer. Undoubtably, targeting the interaction between ferroptosis and lipid metabolism provides potential therapeutic strategies for cancer. The combination of inducing ferroptosis and blocking tumor immune escape may improve the efficacy of antitumor therapy in clinics.
